# A Law of Registration of Births, Marriages and Deaths

**Published:** 1874-01

**Authors:** C. B. Nottingham

**Affiliations:** Georgia


### A LAW OF REGISTRATION OF BIRTHS, MARRIAGES AND DEATHS.

BY C. B. NOTTINGHAM, M.D., OF GEORGIA.

Whilst "too much legislation" is an evil that is deprecated by all good citizens, and although some unnecessary laws may already have a place in our book of statutes, there are nevertheless some subjects underlying the great interests of the people of Georgia, that hitherto have been either totally neglected, or only partially provided for by law, that have strong and daily-increasing claims on the attention of legislators, and should not longer be overlooked; else evils far-reaching and irreparable must inevitably result. Of these subjects, probably there is not one in regard to which appropriate legislation is of more pressing importance, or would be of more extensive and varied usefulness, than that which constitutes the theme of this article. For many years the want and indispensableness of a law providing for the registration of the births, marriages and deaths among our people, has engaged the thoughts of physicians, as well as of other citizens, in different sections of the State; and last Spring, at the annual meeting of the Georgia Medical Association—an organization that has on its roll of membership about six hundred names, embracing much of the intelligence and worth of the profession of the commonwealth—these thoughts took form and character, so far as physicians are concerned, in a determination to approach the government and request the enactment of such a law. At the meeting in Atlanta in April—the largest in the twenty-four years history of the Association—a resolution was adopted under which a committee of seven members was appointed to unite with the Attorney General in perfecting a bill of registration, which is to be presented to the Legislature of this Winter, with a memorial setting forth its claims to consideration, and urging its enactment into law. A simple, cheap bill will be in readiness, and we are hopeful of success in securing its passage.

Registry laws are no new or untried thing. They have existed in the most civilized countries of Europe for a long period, and within the last few years they have been adopted in a number of the most advanced States of this Union. Wheresoever introduced, they have effected great good—have been found practically useful in the advancement of science and the promotion of the public welfare—and peoples who have hitherto neglected them are now earnest and active in an effort to incorporate them among their statutes.

Indeed, when they are considered in their high and sacred relations to property, family and community, they must be adjudged of the highest dignity and of immense value. In the absence of one from the Georgia Code the identification of individuals, the proof of the rightful ownership of property and the evidence of important events in domestic life is even now—in the first half of the second century of our history as a people—sometimes a matter of difficulty or even impossibility, and leads to the most serious embarrassments. If this had been found to be the case thus early it is reasonable to suppose—nay, it can be predicted with unerring certainty, that as the State grows older, generations come and go, the difficulty will be of more frequent occurrence, the embarrassments will greatly multiply and wrong and injustice—moral, social and pecuniary—will be likely to result in many instances and to many persons unless provisions is made by the "law-making power" for the regular, annual gathering, and the placing permanently on record authentic and reliable information of the important events of birth, marriage and death—events that usually control estates, often involve the happiness of families, and to a large extent affect the interests and prosperity of community.

*This reeord* can only be had under the requirements, provisions and authority of general, official registration of the circumstances in the year in which they occur. Memory at best is treacherous, and when the grave questions of lawful wedlock; of legitimacy; of geneological relations; of personal obligations, and of the title to property are in litigation, as is often the case in the courts of justice or are matter of inquiry or in dispute in that other high tribunal, one of the starchambers of society, which is not an incident of rare occurrence—something more than the uncertain and irregular returns of the officiating clergyman or magistrate in regard to marriage—the only one of the three vastly important events concerning which any legislation whatever has been provided—or perhaps the mere recollection of some aged individual in regard to either one of the three several matters, should be readily attainable in determining the weighty interests of heart and purse that may be in jeopardy. But whilst the information that would be obtained on the several subjects embraced in the provisions of the law of registration now sought to be engrafted on the Code, would be valuable to us in common with the great mass of citizens in being emineutly useful in subserving the ends of justice, truth and intelligence, and in the prevention of fraud, villainy and wrong; the *mortuary feature*—that relating to death and its cause—especially would be to us as medical men, the conservators of the public health and students of sanitary science, of greatest interest and highest value. The truthful and reliable expositions furnished from year to year, in regard to the per centage of aggregated and local populations; of the sanitary influence of the pursuits and habits of the people; of the death rate from preventable as well as from unavoidable diseases; of the loss to the State of its true wealth in people from these causes; of the salubrity or insalubrity of the different sections of our territory; of the maladies most prevalent in particular localities; of the limits in which special affections are on the one hand most rife or on the other one least likely to occur, or of districts where persons *suffering from or predisposed* to certain diseases would have the most propitious surroundings of cure or prevention in climate, air and water—the facts faithfully collected on these and kindred subjects would be of incalculable benefit. When aggregated at the office of the Comptroller General they would constitute a mass of statistic information that would open up to the profession of the State material of the profoundest interest for investigation, analysis, comparison and deduction that would edify, instruct and illumine the medical mind—the rich fruits of which would be reaped by all classes of citizens in the improved capacities of their trusted advisers to ward off diseases, effect cures and secure to them the greatest possible amount of health.

To a law of registration there can be under any circnmstances, or from any point in which the matter can be viewed, but one solitary rational objection, and that is the little expense that may attend its execution. This, I apprehend, can be obviated in a great measure by making its provisions few and simple and its machinery simple. In Georgia no new office need be created, nor no new officer created, as no more suitable agents to execute the law can be found than the Tax Receivers of the different counties, under the direction of the Comptroller—these functionaries when receiving the tax returns, if provided with the proper forms and nomenclature, could and doubtless would secure and return very full and complete information from their respective populations, and this without any additional pay to that allowed for the service they now render. If it is doubted, however, that they would be efficient in the discharge of the new-imposed duty without increased remunerative, then let them receive a minimum per capita fee to be paid out of the treasury of their counties under the direction of the Grand Juries of the same. The amonnt thus paid would be so small that it could not be felt as the least burthensome to any class of tax-payers.

Can we not then lay our appeal for a registry law at the feet of the legislators of the commonwealth, with reasonable hope of success? Can it be doubted that the judgment of the good policy of the enactment of the law, expressed last April by that large, patriotic and scientific body—the Georgia Medical Association—seconded as that judgment is by the voice of large numbers of other intelligent and influential citizens—will receive confirmation by those in place and power? Will not the "law-makers" by appropriate legislation at once place Georgia in the front rank of her sister States, in the adoption of a measure that some of the States have already adopted and that all soon will adopt, when the measure will cost comparatively little or nothing; is commended by the sanction of experience, challenges the homage of reason, lays tribute on the best feelings of the heart and is certain to be serviceable in a high degree to the present population and of untold value to the coming generations of our descendants—her people.

Surely disappointment will not be the result of the memorial we are directed to address to the Legislature at its approaching session! An enlightened judgment and far-seeing sagacity will direct their action. Legislation will be had, the bill will be enacted, and suitable measures will be taken to secure the thorough and faithful execution of the law.

